# Cervical Cancer in the Baltic States: Can Intelligent and Personalized Cancer Screening Change the Situation?

**DOI:** 10.15388/Amed.2022.29.1.18

**Published:** 2022-06-29

**Authors:** Mindaugas Stankūnas, Kersti Pärna, Anna Tisler, Anda Ķīvīte-Urtāne, Una Kojalo, Jana Zodzika, Nicholas Baltzer, Jan Nygard, Mari Nygard, Anneli Uuskula

**Affiliations:** Department of Health Management, Lithuanian University of Health Sciences, Kaunas, Lithuania; Institute of Family Medicine and Public Health, University of Tartu, Tartu, Estonia; Institute of Family Medicine and Public Health, University of Tartu, Tartu, Estonia; Institute of Public Health, Rīga Stradiņš University, Riga, Latvia; Institute of Public Health, Rīga Stradiņš University, Riga, Latvia; Institute of Public Health, Rīga Stradiņš University, Riga, Latvia; Cancer Registry of Norway, Oslo, Norway; Cancer Registry of Norway, Oslo, Norway; Cancer Registry of Norway, Oslo, Norway; Institute of Family Medicine and Public Health, University of Tartu, Tartu, Estonia

**Keywords:** cervical cancer, prevention, screening, Estonia, Latvia, Lithuania

## Abstract

The three Baltic States (Estonia, Latvia, and Lithuania) are among the European Union countries with the highest incidence and mortality rates for cervical cancer. In order to tackle this public health challenge, there is an urgent need to implement more advanced and effective methods in cervical cancer prevention in Baltic countries.

Nationwide cervical cancer screening programs in the Baltic States commenced in 2004–2009. While the organized screening programs in these countries differ in some relevant details (target age groups, screening interval), the underlying principles and problems, barriers are universal. However, the outcomes of present screening programs are unsatisfactory. In addition, universal screening programs are extremely costly. There is a potential need for more intelligent and personalized cervical cancer screening program. In 2019 the project *“Towards elimination of cervical cancer: intelligent and personalized solutions for cancer screening”* (2020–2023) was developed with the main objective – to develop improved and personalized cancer screening methods within a sustainable health care system.

It is expected, that more sophisticated cervical cancer screening model will be implemented in Estonia, Latvia, and Lithuania, and will have a positive impact to epidemiology of cervical cancer and public health in general.

## Santrauka.

Trys Baltijos valstybės (Estija, Latvija, Lietuva) yra tarp aukščiausius gimdos kaklelio vėžio sergamumo ir mirtingumo rodiklius turinčių šalių Europos Sąjungoje. Atsakant į šį iššūkį visuomenės sveikatos priežiūros sistemai, yra svarbu taikyti modernesnes ir efektyvesnes gimdos kaklelio vėžio prevencijos priemones Baltijos valstybėse. Nacionalinės gimdos kaklelio vėžio ankstyvosios diagnostikos programos Baltijos valstybėse buvo pradėtos 2004–2009 metais. Nors šalių taikomos programos turi skirtumų (amžiaus grupė, patikrų intervalai), tačiau pagrindiniai principai ir problemos yra panašios. Nepaisant to, dabartiniai šių programų rezultatai netenkina. Juo labiau kad visuotinai taikomos ankstyvosios diagnostikos programos yra brangios. Tai rodo intelektualesnių ir individualizuotų gimdos kaklelio vėžio ankstyvosios diagnostikos programų poreikį. 2019 m. buvo parengtas projektas „Link gimdos kaklelio vėžio suvaldymo: intelektualūs ir individualizuoti sprendimai ankstyvajai vėžio diagnostikai“ (2020–2023), kurio pagrindinis tikslas yra sukurti tobulesnius ir individualizuotus vėžio ankstyvosios diagnostikos metodus tvarioms sveikatos priežiūros sistemoms. Tikimasi, kad projekto metu parengtos pažangesnės gimdos kaklelio vėžio ankstyvosios diagnostikos rekomendacijos bus taikomos Estijoje, Latvijoje ir Lietuvoje ir taip prisidės prie teigiamų pokyčių gimdos kaklelio vėžio epidemiologijoje ir apskritai visuomenės sveikatai.

## Introduction

Cervical cancer (CC) is the fourth most common cancer in women worldwide, responsible for approximately 570 000 cancer cases and 311 000 deaths annually [1]. The three Baltic countries (Lithuania, Latvia, Estonia) are among the European Union countries with the highest incidence and mortality rates for CC [2]. Previous studies have revealed that this situation can be explained by a comparatively lower quality of disease-preventative health care services [3] in the Baltic region resulting in a largely opportunity-based screening approach with low population-level coverage and suboptimal quality of available screening tests [4]. With major advances in our understanding of the infection etiology of CC and its drivers, new tools and possibilities in preventive medicine are emerging [5]. Human papillomavirus (HPV) vaccines, together with a growing arsenal of HPV-based screening tests, offer enormous promise for control and ultimately elimination of the CC progression pathway. The modeling study by Vaccarella (2016) estimated that national programs for screening of the CC could save more than 9000 lives till 2040 in the Baltic States (Lithuania – 4783; Latvia – 2770, and Estonia – 1461) [6]. This emphasizes that CC is a major public health concern in Estonia, Latvia, and Lithuania, that needs urgent action.

The population size of the Baltic region (Lithuania, Latvia, and Estonia) is a little bit over six million. Women aged 25–69 years comprise almost one-third of the population (1.89 million). Disparities in CC incidence and mortality rates between Baltic countries and Scandinavian/Western European are well documented and long-standing.

Estimated incidence (age standardized) of CC for 2020 varies among the countries: Estonia – 18.5 per 100,000; Latvia – 18.4, and Lithuania – 18.7, with the European Union average at 12.8 [7]. Similar differences can be observed in mortality rate statistics. The estimated standardized mortality rate from CC for 2020 in Estonia was 4.3 per 100,000, 6.8 per 100,000 in Latvia, and 6.7 per 100,000 in Lithuania with the European Union average at 5.3 per 100,000 [7]. Closing this health gap requires decisive action.

## Policy options

Identifying alternative policy option is, in most cases, an iterative process. The aim is to consider and evaluate realistic alternatives as possible and then narrow them down to the most relevant ones to be tested and implement in case of grounded justification.

### Prevailing policy option

Until now, cervical cytology based (Pap test) cancer mass screening is the principal strategy and has considered as one of the 20th century’s success stories. The initial success [8] has, however, been followed by stagnation which has multiple causes [9-12]. Most importantly this approach has not lead into expected public health benefits in many locations including the Baltic countries.

Before the declaration of independence in 1991, Lithuania, Latvia, and Estonia, were part of the Soviet Union, which used the Semashko health care system. This system had a minimal and primitive approach to disease prevention and health maintenance. Major reforms of the national health systems were initiated in late 90s and accelerated by acceding to the EU in 2004 along with the associated financial and technical assistance that became available.

A pilot program of organized CC screening in Estonia started in 2003 [13]. Screening invitations were sent to 12,960 randomly selected women aged 30 to 40 years with health insurance, about 22% responded (had Pap test taken in response to the invitation) and close to 7% had abnormal cytology (Pap test) findings. In 2006, nation-wide program was initiated, and organized via screening cabinets in clinics that participated in the program with specially trained midwives taking the cytological tests [14]. Women were invited for screening using individual invitation letters sent by e-mail, by post or via the media information campaigns (the exact methodology differs by year). Beginning in January 2021, Estonia implemented new guidelines recommending primary HPV testing for women aged 30 to 65 years at the five-year intervals [15]. The National Health Development Institute under the Ministry of Social Affairs of Estonia operated the program.

In Latvia, organized CC screening started in 2009 and the main responsibility was delegated to general practitioners (GP). CC screening was implemented utilizing three approaches to access women: 1) National Health Service sends invitation to participate in CC screening to all target age women; 2) a woman visits the GP due to any medical condition and she is advised to have a Pap test; 3) gynecologists or obstetricians use any visit (e.g., for birth control counseling) for advising women to have cytological testing. Over the first three years of screening program operation the coverage rose from 15% to 35% [16].

The Lithuanian national screening program was launched in 2004, using conventional Pap test along with a modified Bethesda cytological classification as a screening test. The National Health Insurance Fund under the Ministry of Health of Lithuania finances the program. Primary health care centers are responsible for carrying out the invitations and performing Pap tests. Usually personal invitations are not sent out by mail and GP tend to rely on informing women about the screening when they attend their primary health care center [17, 18]. The program still carries opportunistic features because it is strongly dependent on the frequency of visits to the GP and the activity of the GP in providing information about screening [19]. Data on the exact coverage of screened women is currently not available. Research projects testing the effect of personal invitation letters conducted in 2011 and 2014 in Lithuania yielded response rates (coverage) from 22% [17] to 25% [18]. Nationwide CC programs commenced in 2004–2009. The target age groups and screening intervals differ by country with the most intensive screening model implemented in Latvia (Table 1). CC screening registries are established in Latvia (since 2009) and Estonia (since 2016) [20], but not in Lithuania [21].

Until 2020, cytology was the primary screening test in all three Baltic States (in Estonia, beginning from 2021, HPV DNA test is in use). All of the three countries lack comprehensive screening test quality control system. In addition, the specific methodology for CC specimens staining differs between countries and from the traditional multichromatic (five stains in three solutions) cytological staining technique developed by George Papanicolaou. Instead of the Papanicolaou cytological tests (Pap tests), recommended by the European guidelines [22] the technique used in assessing cytological smears in Latvia is Giemsa stain in Leishman modification, a unique historical tradition in the former Soviet Union cytology [23]. Local research has postulated inadequate screening uptake and insufficient quality of the Pap test based screening program as drives behind the failure of CC prevention [24]. The comparatively low uptake in Latvia has been explained by two factors [16, 24]. Firstly, cytological testing outside the program is still very frequent and performed on an ongoing basis in parallel with the organized screening [16]. Secondly, low participation rate could be related to Latvian women poor understanding of the roles that cervical screening and HPV vaccination play in preventing CC [25].

**Table 1.** Overview of cervical cancer prevention, population demography and cervical cancer epidemiology in the Baltic States (as of December 2020).

Indicator	Lithuania	Latvia	Estonia
***Cervical cancer screening***			
Introduction of organized screening	2004	2009	2006
***Organized screening implementation***			
Nationwide	2004	2009	2006
***Screening guidelines until 2019***			
Primary screening test	Pap test (cytology)^1^		
Screening target ages, and frequency	29–59 yr. old, 1 test / 3 years	25–69 yr. old, 1 test / 3 years	30–55 yr. old,^2^ 1 test / 5 years
Screening coverage (%)	53.8 (2018)	39.7 (2019)	50.9 (2017)
***HPV vaccination***			
Program for adolescents (year and target population)	Since 2016, 11 yr. old girls	Since 2010, 12–18 yr. old girls	2018, 2019 12–14 yr. old girls; 2020 12 yr. old girls only
HPV vaccination program coverage (%)	na	69.2% (2019)	35%
***Cervical cancer epidemiology (latest available)***			
Age-standardized (World Standard Population) incidence rates per 100 000 women-years	20.4 (2008)	14.3 (2018)	12.0 (2018)
Cum. inc. per 100 000 women-years by age 75 years	na	1.4 (2018)	2.2 (2018)
Annual number of new CC cases	420 (2020)	216 (2018)	127 (2018)
Annual number of CC-related deaths	189 (2018)	125 (2019)	63 (2019)
1-year relative survival (95% CI)	77% (76 – 79) ^3^	75% (73 – 73)^3^	84% ^4^
5-year relative survival (95% CI)	56% (54 – 58)^3^	51% (48 – 54)^3^	67% (64 – 70) ^4^

1 HPV DNA test beginning from 01.01.2021 in Estonia.

2 30–65 years beginning from 01.01.2021 in Estonia.

3 For years 2001–2007.

4 For years 2010–2014. For years 2004–2004 1-year relative survival 84%, 5-year relative survival 64% (60–68).

### An alternative policy option

Challenges of mass-screening, with its one-size-fits-all strategy, at micro-level (low awareness and knowledge about CC, lack of access to information, low risk perceptions, and poor health seeking behaviors), meso-level (social networks, socio-cultural norms) and macro-level barriers (costs of screening, lack of or nonfunctioning national cancer prevention policies and programs, nonoperational quality assurance systems) are noteworthy.

More effective and cost-effective personalized screening programs is highly relevant. The introduction of HPV vaccines, the use of HPV testing in screening, and the use of HPV typing and other biomarkers after a positive screening test makes a personalized risk assessment pertinent. The concept of personalized cancer prevention is attracting increasing interest as the screening, diagnostic and treatment choices are increasing due to scientific discoveries. The added complexity requires computerized assistance for proper management of population segments with different risk levels (i.e. women infected with HIV) and challenges the conceptual and logistical framework of delivering the existing cancer screening which is designed to deliver preventive health care in a “one-size-fits-all” approach. Personalization beyond HPV status could lead to longer or shorter screening intervals for certain women. Additional risk factors for CC have been identified, but are not currently included in recommendations about screening intervals. These include age, smoking, oral contraceptive use, and sexual history [26]. Evaluating screening strategies that consider these risk factors for a more personalized approach, incl. tailoring screening intervals, are of significant research need.

There is increasing interest in risk-based/stratified screening. Risk stratification models offer a tailored approach to suit each woman’s individual risk [27-30]. Risk-based screening strategy that could help to direct efforts at those most likely to benefit (most at risk) and tailor the screening activities based on risk. Evidence originating from risk-based screening of breast cancer indicates that not offering breast cancer screening to women at lower risk could improve the cost-effectiveness of the screening program, reduce over-diagnosis, and maintain the benefits of screening [30].

There are two main considerations for implementing a more personalized approach to (cervical) cancer screening: i) the risk factors for developing cancer should be known, and ii) data allowing risk identification/stratification should be accessible. CC is a good candidate for risk-based screening feasibility and effectiveness testing.

### Implementing personalized cancer screening in the Baltic States

In 2018 the Baltic States established the Baltic Research Program in response to common challenges in the research sector [31]. This program supports collaborative research projects between the Baltic countries and Norway, Iceland, and Liechtenstein, and is funded by joint collaborations among EEA, Norway Grants, and Governments of the Baltic States. In 2019, the University of Tartu, Rīga Stradiņš University, Lithuanian University of Health Sciences, and the Norwegian Cancer Registry began the project *“Towards elimination of cervical cancer: intelligent and personalized solutions for cancer screening”* (2020–2023).^1^ The project’s main objective is to develop improved and personalized cancer screening methods within a sustainable health care system. The developed methods will integrate knowledge of biological disease mechanisms and available data from national population-based health registries, health care provision data, surveys, and Estonian genome bank to develop, validate, and determine the cost-effectiveness of specific artificial intelligence technology for the purpose of preventive medicine in CC. The foundation of this project relies on combining population based multi-faceted individual data (HPV-status, health and reproductive behavior information; individual histories of CC screening; genetic data) with the advantages of high-performance computing and analytics to leverage existing knowledge and experience for transforming cancer screening systems towards higher inclusiveness as well as making them increasingly flexible, scalable and sustainable.

## Discussion

There is a clear need to modernize and develop CC prevention efforts in all three Baltic countries. Incidence rates of CC are already high in the Baltic region, and are projected to increase over the coming decades. Mass-screening, with its one-size-fits-all strategy, is an infrastructural challenge not only in low resource settings but also in high-income countries, and refining this model to account for individual variation and need will save both lives and resources. Creating a knowledge base for more effective and cost-effective personalized screening is highly relevant for everyone from tax payers to health care professionals. Therefore, we are recommending the following measures in tackling these problems in the Baltic States:

1. *Building public awareness.* Health authorities and stakeholders should invest in effective communication about the effectiveness and safety of both CC screening and HPV vaccines to generate a basis for confidence. Population based programs rising awareness and knowledge about CC especially on opportunities of cancer prevention, and targeting low risk perceptions together with the poor health seeking behaviors are needed.
2. *Developing robust and accountable programs.* Further developing robust system of program management and coordination. The program should assure implementing effective quality assurance strategies across the whole span of the screening (detection, diagnosis, treatment):
  - Establishment of screening registries and linkage of individual screening data with cancer registry data, as essential tools of monitoring and evaluation.
  - Development of Population based systematic screening strategies.
  - Transition to HPV based screening test. This is based on extensive evidences demonstrating higher sensitivity and accuracy, lower variability and better reproducibility of HPV based screening compared with conventional cytology [32]. The primary HPV screening is implemented in Latvia (from July 2022) and Lithuania (from January 2022).
  - Deemphasizing opportunistic screening due to low coverage and over-screening of minority of women.
3. *Creation innovative open programs.* The program design must permit evaluation of new strategies for screening implementation and costs calculations. There is a comparative paucity of robust evidence about the effects of innovations in real world as opposed to experimental or research settings. This can lead to a divergence between the benefits found in experimental settings from those in public health practice.

## Footnotes

1 More information about the progress and results of the project can be find here: https://screeningsaveslives.eu/
